# Double hit of foetal growth restriction and postnatal hyperoxia alters lung structure and function in a preterm rabbit model of bronchopulmonary dysplasia

**DOI:** 10.1371/journal.pone.0330717

**Published:** 2025-08-26

**Authors:** Marnel Greyling, Yannick Regin, Emilie Goffinon, Francesca Stretti, Tomohiro Arai, Giorgio Aquila, Francesca Ricci, Jaan Toelen

**Affiliations:** 1 Woman and Child Division, Department of Development and Regeneration, KU Leuven, Leuven, Flemish Brabant, Belgium; 2 Neonatology and Pulmonary Rare Disease Unit, Experimental Pharmacology and Translational Science Department, Corporate Pre-Clinical Research and Development, Chiesi Pharmaceuticals, Parma, Emilia-Romagna, Italy; 3 Department of Paediatrics, UZ Leuven, Leuven, Flemish Brabant, Belgium; Kobe University Graduate School of Medicine School of Medicine, JAPAN

## Abstract

Bronchopulmonary dysplasia (BPD) is a disease with a multi-factorial pathophysiology; however, current animal models lack complexity. We employed a double-hit model with an antenatal insult of foetal growth restriction paired with milder postnatal hyperoxia exposure. We induced foetal growth restriction (FGR) by injecting N(G)-nitro-L-arginine methyl ester (L-NAME) in the pregnant rabbit, and exposed preterm-born kittens to 70% hyperoxia for 7 days. L-NAME effectively induced FGR, and mortality rates were acceptable. The double-hit group exhibited adverse outcomes, including decreased lung compliance, increased airway resistance, and structural changes such as alveolar simplification and thickened septa. Gene expression analysis in the L-NAME group revealed downregulation of vascular growth factors, suggesting impaired vascular development. In contrast to traditional hyperoxia models, our double-hit approach enables lower hyperoxia exposure, aligning more closely with clinical practice guidelines in neonatology. The findings underscore the importance of antenatal factors in BPD pathophysiology and reinforce the need for refined animal models that accurately reflect the complexities of preterm lung development.

## Introduction

Bronchopulmonary dysplasia (BPD) is a chronic lung disease affecting preterm infants, with the highest risk among those born at the lowest gestational ages and weights [1]. Despite advancements in the management of preterm neonates, the incidence of BPD remains unchanged [2]. Modern management strategies enable the survival of extremely preterm infants, but their underdeveloped lungs make them particularly susceptible to BPD. The phenotype and pathology seen in BPD have evolved from severe fibrosis and emphysema caused by mechanical ventilation and oxygen toxicity to a phenotype marked by impaired alveolar and pulmonary vascular development [3].

BPD pathophysiology is complex, involving antenatal insults, therapeutic interventions, and postnatal complications, all contributing to lung injury [1]. Current animal models typically expose neonates to high oxygen levels to induce BPD-like changes. However, this approach diverges from clinical practice, where the lowest effective oxygen concentration is used to reach target saturations.

One of the important independent risk factors for developing BPD is foetal growth restriction (FGR). FGR is characterised by failure to achieve genetic growth potential [4]. Global BPD trends show that infants born at the lowest weights have exceptionally high rates of BPD [5]. Although FGR has multiple risk factors, the most common cause is placental insufficiency, resulting in oxygen and nutrient deprivation [4,6]. Many FGR animal models have been used in past research, but it is not often combined with postnatal insults in translational BPD research.

The rabbit is an ideal model for studying BPD due to its lung development parallels with humans [7]. FGR can be induced pharmacologically by using L-NAME, a nitric oxide synthase inhibitor that effectively reduces foetal growth but has historically been avoided in preeclampsia research due to placental transfer of active metabolites [6,8]. Nitric oxide (NO) plays a crucial role in alveolarization, and disruptions in NO signalling have been implicated in numerous chronic lung diseases. Using L-NAME to model BPD adds translational value by mimicking antenatal and postnatal risk factors [9].

A “double-hit” model incorporating antenatal FGR and postnatal hyperoxia may improve translational relevance. The canalicular phase of lung development gestational day (GD) 23 - GD27 in rabbits) is a critical window for alveolar formation, aligning with the developmental stage of extremely preterm human infants [7]. However, as rabbit neonates born before GD28 do not survive due to respiratory insufficiency, introducing an antenatal insult during the canalicular phase may enhance understanding of BPD pathogenesis.

Standard rabbit models use extreme hyperoxia, contrasting with clinical practice, where lower oxygen levels are targeted [7]. Other researchers have also focused on expanding the rabbit model with multiple insults in a long-term model [10]. We hypothesise that combining antenatal FGR with moderate hyperoxia in preterm rabbits in a short-term model will create a more physiologically relevant BPD model.

## Materials and methods

### Ethics

All animal experiments were approved by the ethics committee of the Animal Research Centre of KU Leuven under project number P063/2022.

### Animal protocols

Time-mated New Zealand White rabbits were sourced from a recognised breeder through the KU Leuven animal facility. Mothers were housed in a humidity and temperature-controlled room with a 12-hour light/dark cycle and free access to food and water.

**Fig 1:**
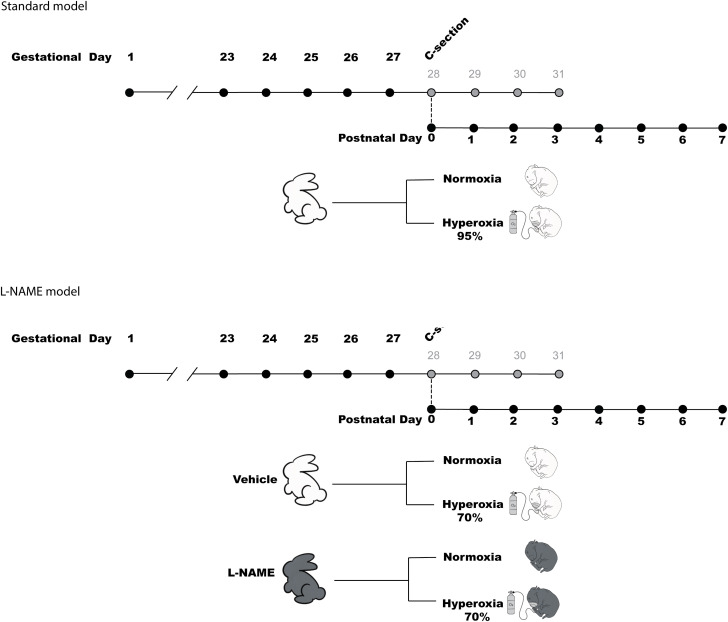
Visual overview of the standard hyperoxia model compared to the double-hit model.

On GD24–27, pregnant does were randomly assigned to receive either L-NAME 62.5 mg/kg (Merck Life Sciences BV, Belgium) or vehicle (saline) by subcutaneous injection (Fig 1).

Lecarpentier et al. studied different doses and timings of L-NAME injection. The dose of 62.5 mg/kg injected on consecutive days during late pregnancy was effective in inducing foetal growth restriction while limiting foetal death [6,11]. The timing of the injections correlates with the canalicular phase of lung development.

On gestational day 28 (term: GD31), mothers were sedated with ketamine 20 mg/kg (Nimatek, Eurovet Animal Health, Nederland) and xylazine 5 mg/kg (XYL-M 2%, V.M.D. n.v., Arendonk, Belgium) as an intramuscular injection. Once sedated, mothers were euthanised by injecting T61 1mL (Embutramide 200 mg, Mebenzonium iodide 50 mg, Tetracaine hydrochloride 5 mg, MSD Animal Health, Brussels, Belgium) into the marginal ear vein. Once the absence of a corneal reflex confirmed death, the kittens were delivered by rapid caesarean section. A lower midline incision was made, and the bicornuate uterus was exposed. Kittens were removed one by one after hysterotomy, and their membranes were manually removed before being dried, stimulated, labelled, and placed into a temperature- and humidity-controlled incubator (temperature: 32°C, humidity: 50%). An hour after birth, kittens were weighed, measured, and randomised to either normoxia (Nox) or 70% hyperoxia (Hox) for 7 days.

Kittens were stimulated to urinate twice daily, followed by feeding via orogastric tube. Kittens were measured at two time points (birth, postnatal day (PND) 7) and weighed daily in the morning before feeding. The length was measured from the fontanel to the base of the tail with the kitten lying on its side. Feeds consisted of a milk replacer (Day One, Protein 30%, Fat 50%; Fox Valley, Illinois, USA) fortified with immunoglobulins and probiotics (Col-o-Cat, SanoBest, Hertogenbosch, Netherlands) for the first 2 days, followed by fortification of milk feeds with probiotics alone (Puur Probiotic, NML Health, Netherlands) until harvest. Feed volume was calculated as a percentage of body weight, and gradually increased in the first 4 days of life.

### Pulmonary function testing

On PND 7, kittens were sedated with ketamine 20 mg/kg and xylazine 5 mg/kg by intraperitoneal injection. This sedation was terminal. Once kittens were sedated, a tracheotomy was performed, and a cannula was secured with an air-tight suture. The cannula was connected to the Scireq Flexivent system, and invasive pulmonary function testing was performed by the broadband forced oscillation technique. With this method, ventilation of the subject is temporarily paused, a test signal is delivered to the lungs, and the response is measured. Based on a mathematical model, the response of the lungs can be separated into that of the central airways and that of the lung parenchyma. Two important parameters extrapolated are tissue compliance and tissue elastance. Compliance is an indication of the ease with which a respiratory system can be distended. The lungs require elasticity to be able to extend sufficiently to move air in and out of the lungs during tidal breathing. Tissue elastance (H) is an indicator of alveolar stiffness, representing the elastic energy stored in the lung tissue after deformation, and thus the tissue’s ability to revert to its original shape after imposed distension. Tissue damping (G) gives an indication of peripheral airway and alveolar resistance by extrapolation of the amount of energy lost in the peripheral lung tissue due to friction.

### Histological analysis

Once pulmonary function testing was completed, the lungs were harvested for histological and molecular analysis. In short, the deeply anaesthetised kittens were euthanised and the lungs were dissected *en bloc* before separating the right and the left lungs. Harvesting and fixation of the lungs were done as previously described [12–14]. In short, the right lung was flash-frozen in liquid nitrogen, and the left lung was fixated for at least 24 hours with 4% paraformaldehyde at a constant hydrostatic pressure of 25 cmH_2_O. Once fixation was complete, lung volumes were measured by immersion in saline, before embedding in paraffin [15].

Lungs were sectioned into 5-micrometre sections, and haematoxylin and eosin staining was performed on two sections per lung. Stained sections were scanned using the Axio Scan Slide Scanner (Zen Zeiss, Oberkochen, Germany). At least 30 random regions of interest were selected per lung section by using an algorithm in QuPath V0.3.2. Alveolar morphological analysis was performed using a semi-automated method for unbiased analysis. Reference points and intercepts were used to determine the mean linear intercept (Lm) and mean transsectional wall length (Lmw) [16]. The total surface area of the airspaces and total lung volume attributed to alveolar septa were calculated as a measure to evaluate alveolar development. A decreased surface area and total volume of septa would correlate with decreased alveolar numbers/alveolar simplification. The medial thickness of pulmonary arterioles was calculated after measurement of the internal and external diameter of at least 30 vessels on 5-micrometre lung sections that were Miller’s elastin-stained. Vessels with an external diameter outside the range of 20–100 micrometres were excluded. A single, blinded investigator performed all histological analyses.

### Gene expression

Total RNA was extracted from flash-frozen lung tissue using Tripure Isolation Reagent (Roche Life Science, from Sigma-Aldrich, Belgium). cDNA was synthesised using a High-Capacity Reverse Transcription Kit (Applied Biosystems, Thermo Fischer Scientific, Belgium). Specimens for qPCR were run in triplicate and expression of VEGFA and eNOS was detected using PowerUp SYBR Green Master Mix (Applied Biosystems, Thermo Fischer, Belgium). Gene expression was normalised to the expression of the HPRT housekeeping gene. The comparative threshold cycle method was used for quantification and statistical analysis of results. Primers were ordered from Integrated DNA Technologies IDT, Belgium. Primer sequences are listed in the supporting information (S1 Table).

### Statistical analysis

For sample size calculations, the effect size was calculated using the static compliance of normoxic animals from previous studies conducted by our group. A Bonferroni correction for alpha was applied before performing the ANOVA. A sample size of 12 animals per group would provide 80% power to detect a 20% difference in static compliance.

GraphPad Prism 10 (GraphPad Software, Inc., Boston, USA) was used for statistical analysis. A log-rank test was used to compare survival. The Shapiro-Wilk normality test was performed to determine the Gaussian distribution. For data on postnatal day 0, unpaired t-tests were performed. For other data, one-way ANOVA and Dunnett’s multiple comparison test were performed to compare single and double hits with the control and normoxia. Where multiple measurements were done, a mean was calculated and represented as a single data point. Values are expressed as mean ± standard deviation. A p-value of p < 0.05 was considered statistically significant.

## Results

**Fig 2:**
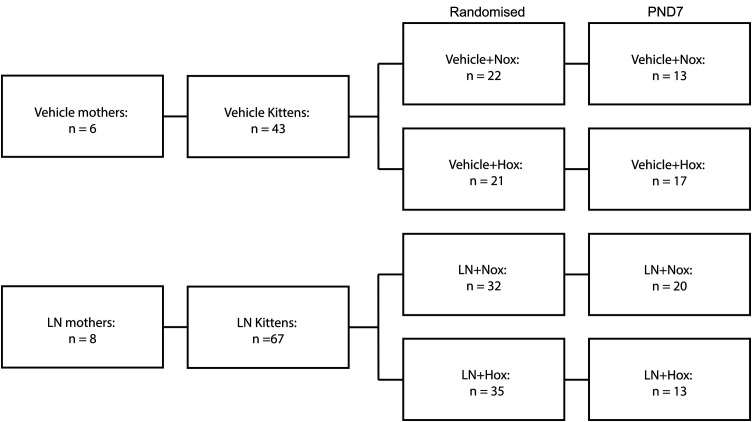
Overview of the number of animals used for statistical analysis. n: number, Nox: normoxia, Hox: hyperoxia, Veh: vehicle.

Kittens were randomized into four groups: Veh + Nox (Vehicle + Normoxia), Veh + Hox (Vehicle + Hyperoxia), LN + Nox (L-NAME + Normoxia), LN + Hox (L-NAME + Hyperoxia). Kittens from 14 mothers were used across all experiments (Fig. 2).

### Survival

**Fig 3:**
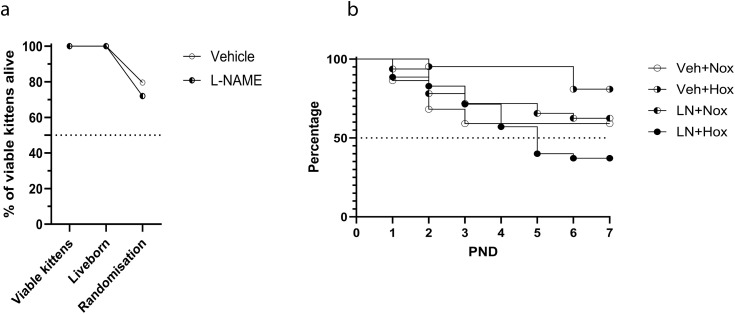
Survival of kittens to (3a) delivery and randomisation and (3b) from randomisation to postnatal day 7. PND: postnatal day, Veh + Nox: vehicle + normoxia, Veh + Hox: vehicle + hyperoxia, LN + Nox: L-NAME + normoxia, LN + Hox: L-NAME + Hyperoxia.

In a model that includes an antenatal as well as postnatal insult, there are several critical time points for survival: survival to delivery (i.e., foetal death), survival from birth to randomisation after 1 hour, and survival to postnatal day 7. A model with excessive mortality would be unacceptable as a translational model. Foetal death rates were low, and survival to randomisation was acceptable. Antenatal injection of L-NAME did not cause significant foetal deaths (Fig 3a). The LN + Hox kittens had significantly lower survival to PND7 (37%) (Fig 3b). The cause of death was not able to be determined in the majority of cases, with a few dying due to complications during feeding. The unexplained deaths likely represent the more severe phenotype of the model.

### Foetal growth restriction was evident at birth, and hyperoxia impacted postnatal growth

**Fig 4:**
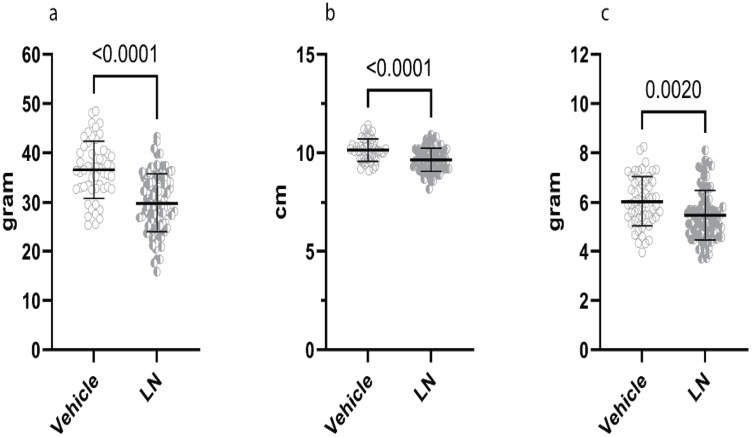
Anthropometry at birth, (4a) weight, (4b) length, (4c) placental weight. Veh + Nox: vehicle + normoxia, Veh + Hox: vehicle + hyperoxia, LN + Nox: L-NAME + normoxia, LN + Hox: L-NAME + Hyperoxia.

Foetal growth was assessed by measuring and weighing kittens at birth and assessing placental weight. Kittens exposed to L-NAME were significantly lighter at birth compared to those in the control groups. L-NAME kittens were 20% lighter at birth compared to control kittens (mean weight 29.85g vs 36.60g) (Fig 4a). The mean length of L-NAME kittens was 7.4% shorter than that of controls (9.65 cm and 10.14 cm, respectively) (Fig 4b). Placental weight was 9% smaller in the L-NAME group when compared to controls (Figure 4c).

**Fig 5:**
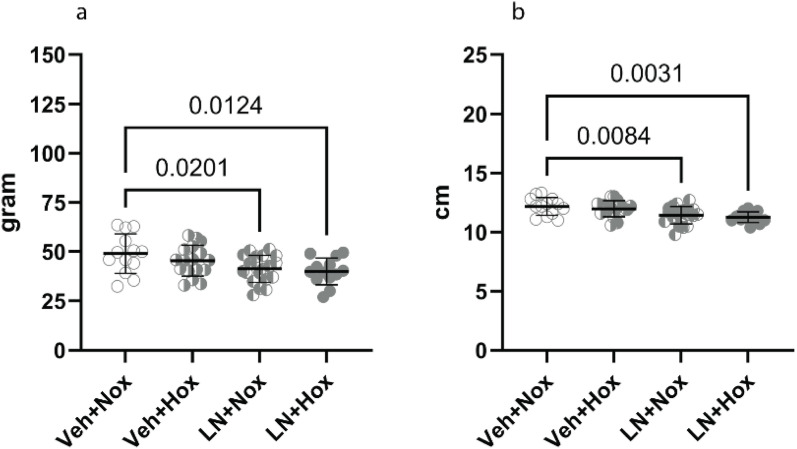
Anthropometry at postnatal day 7, (5a) weight, (5b) length. Veh + Nox: vehicle + normoxia, Veh + Hox: vehicle + hyperoxia, LN + Nox: L-NAME + normoxia, LN + Hox: L-NAME + Hyperoxia.

Weight and length measurements were repeated on postnatal day 7 to assess catch-up growth. Kittens who were growth-restricted at birth remained lighter at PND7 compared to controls. Control kittens who were of normal weight at birth, but randomised to hyperoxia, were lighter at PND7 compared to vehicle-normoxia kittens (Fig 5a). L-NAME kittens were still shorter than controls at PND7 (Fig 5b).

### A double hit negatively affects pulmonary function

**Fig 6:**
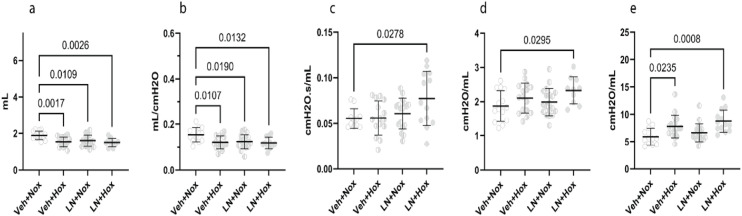
Pulmonary function tests: (6a) inspiratory capacity, (6b) static compliance, (6c) Newtonian resistance, (6d) tissue damping, (6e) tissue elastance. Veh + Nox: vehicle + normoxia, Veh + Hox: vehicle + hyperoxia, LN + Nox: L-NAME + normoxia, LN + Hox: L-NAME + Hyperoxia.

Multiple parameters were affected negatively in the LN-Hox group. Inspiratory capacity (Fig 6a) (p = 0.0026) and static compliance (Fig 6b) (p = 0.0132) were decreased. Newtonian resistance (Fig 6c) (p = 0.0278), tissue damping (Fig 6d) (p = 0.0295), and elastance (Fig 6e) were increased (p = 0.0008).

### A double hit induced structural pulmonary changes

**Fig 7:**
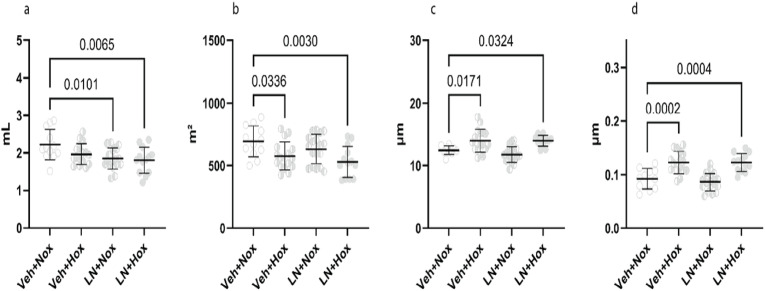
Histological analysis for structural lung changes: (7a) lung volume, (7b) surface area of air spaces, (7c) mean transsectional wall length, (7d) pulmonary artery medial thickness. Veh + Nox: vehicle + normoxia, Veh + Hox: vehicle + hyperoxia, LN + Nox: L-NAME + normoxia, LN + Hox: L-NAME + Hyperoxia.

Lung volume (Fig 7a) was reduced in the double-hit group (p = 0.0065). The absolute surface area of the air spaces (Fig 7b) was reduced (p = 0.0030). The septal thickness based on mean transsectional wall length (Fig 7c) was increased in the double-hit kittens (p = 0.0324). As a measure of pulmonary hypertension, the thickening of the medial thickness of pulmonary arteries (Fig 7d) was observed in the double-hit group (p = 0.0004). These findings were consistent with those of BPD, characterised by decreased alveolar volume, thickened septa, and a negative impact on pulmonary vasculature.

### L-NAME with and without hyperoxia, reduced expression of vascular genes

**Fig 8:**
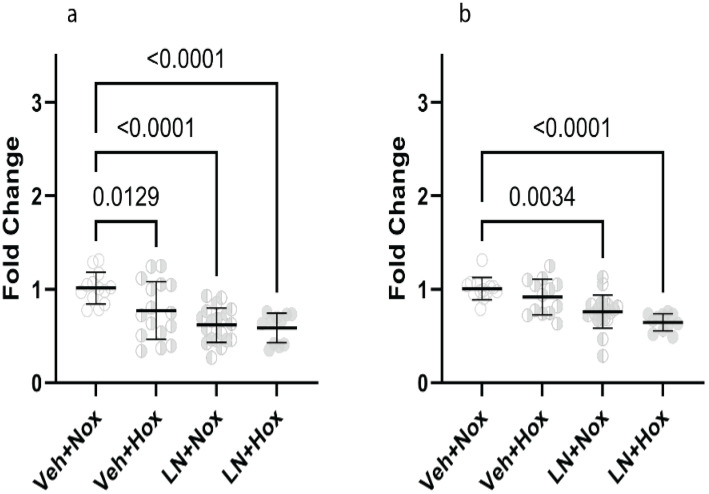
Gene expression in lung tissue: (8a) eNOS, (8b) VEGFA. eNOS: endothelial nitric oxide synthase, VEGFA: vascular endothelial growth factor A, Veh + Nox: vehicle + normoxia, Veh + Hox: vehicle + hyperoxia, LN + Nox: L-NAME + normoxia, LN + Hox: L-NAME + Hyperoxia.

We performed qPCR on the lung samples from the LN experiment to gain a better understanding of gene expression. In all LN kittens, the expression of eNOS (Fig 8a) was decreased (p < 0.0001). VEGFA (Fig 8b) expression decreased in all LN kittens, but to a lesser extent in the LN + Nox kittens than the LN + Hox kittens (p = 0.0034 and p < 0.0001, respectively).

## Discussion

The double-hit model effectively induced both structural and functional changes in the lungs consistent with BPD. In our research group, various methods for inducing foetal growth restriction (FGR) were tested; however, antenatal administration of L-NAME had the most favourable mortality rates and produced a phenotype aligning with BPD. This supports the concept of foetal programming and opens avenues for interventional or therapeutic studies.

BPD is a disease with complex pathophysiology and a wide range of phenotypes. With advances in perinatal care, infants born at the extremes of viability are now able to survive, yet the prevalence of BPD remains high. A recent meta-analysis showed that BPD prevalence increases with lower gestational age, 43% in extremely low gestational age versus 12% in very low gestational age. It also revealed a strong correlation with birth weight, independent of gestational age, with rates reaching 71% for infants born weighing less than 500g [2].

Although BPD often has devastating effects on patients and their families, most infants now survive, complicating the scientific study due to the limited availability of histopathological samples for analysis. Animal models, therefore, remain of high importance.

The rabbit model has been well studied in the context of BPD research. In our group, this involves delivering rabbit kittens on gestational day 28 (3 days before term), when they enter the saccular lung development stage. For the first seven days of life, kittens are exposed to 95% oxygen (hyperoxia) to induce lung injury, resulting in structural and functional changes that mimic those of BPD [7,13]. A major critique of this model is its deviation from clinical practice in terms of the use of oxygen. In clinical neonatology, oxygen concentration is guided by targeted saturation monitoring to limit oxygen-induced lung injury. Another difference is the absence of multiple exposures or events that could impact lung development and health. Preterm neonates are not only at risk of developing BPD due to preterm birth and the necessary life-saving interventions and often unavoidable complications, but also by antenatal factors that are frequently the inciting event for preterm birth. Thébaud et al. hypothesised that multiple antenatal and postnatal insults could activate different pathways in the developing lung, resulting in diverse and complex lung injuries [17]. This hypothesis is supported by a study by Higano et al. where MRI images were analysed and found that different regions of the lung in BPD patients displayed heterogeneous pathology, from decreased alveolarization to fibrosis, emphysematous changes and airway injury [18]. The phenotypes of BPD are complex, with different anatomical compartments being affected in various ways in different patients [19]. Pulmonary function testing in infants confirms that not all patients have obstructive lung disease; isolated restricted lung disease and a combination of both can be found in BPD patients [20]. Considering the complexity and variety in phenotypes of BPD, our hyperoxia rabbit BPD model is overly simplistic and does not mimic the multiple insults a preterm-born infant is more often than not exposed to.

Many infants born preterm and who go on to develop BPD are born growth restricted [2]. Foetal growth restriction is characterised by a foetus failing to reach its genetic growth potential, often resulting in infants who are small for gestational age and have low birth weight [4]. There is increasing recognition of foetal programming or the developmental origins of adult diseases, with prenatal insults considered sufficient to induce sustained structural changes in lung development [17,21]. FGR is one such example and is recognised as an independent risk factor for developing BPD [22,23]. Quantifying the global incidence of FGR is challenging due to differing pathophysiology, definition and diagnostic criteria across locations. The estimated incidence ranges from 5–10% in developed countries to 20–30% in developing countries [24,25]. Maternal (smoking, malnutrition, pregnancy-associated diseases), placental and foetal (congenital diseases, chromosomal abnormalities) factors can cause FGR, with placental insufficiency being the primary cause in developed countries [4,26]. In our study, FGR-kittens had a higher mortality rate, especially in the double-hit group, suggesting a predisposition to further injury caused by the initial insult, and supporting the foetal programming hypothesis.

Abnormal placental vascular structure identified on histopathological analysis in placental hypoperfusion is strongly associated with FGR. In such pregnancies, infants are not only more susceptible to BPD but also to pulmonary hypertension. Mestan et al. and Check et al. also showed that unfavourable intrauterine events negatively impact the growth of pulmonary vasculature, predisposing FGR infants to BPD and pulmonary hypertension [27,28]. Multiple animal models have been used to study FGR, many of them in rabbits [26]. The rabbit has an advantage over the rodent (rats/mice) due to its larger size, which makes performing procedures technically less challenging. Rabbits also have a short gestational age (term: 31 days) and large litter sizes. Given our group’s experience with the preterm rabbit model, incorporating an antenatal FGR insult in pregnant rabbits was feasible.

Endothelial-derived nitric oxide synthase (eNOS), is an enzyme that combines L-arginine with oxygen and the cofactor NADPH to produce L-citrulline and nitric oxide [29]. NO has various biological effects, including smooth muscle relaxation and vessel dilation, as well as angiogenesis and vascular remodelling, along with some anti-inflammatory and antithrombotic activity [29]. When eNOS activity or expression is reduced, it leads to vasoconstriction, smooth muscle proliferation and increased pulmonary resistance [29–31]. NO also mediates vascular endothelial growth factor (VEGF)-induced lung angiogenesis, and in turn, VEGFA stimulates eNOS activity via a specific signalling pathway [32]. VEGFA is part of a family of genes that mediate angiogenesis by stimulating endothelial cells to proliferate and migrate via either a sprouting or an intussusceptive mechanism. VEGFA is not only responsible for developmental angiogenesis, but also for pathological angiogenesis, with disrupted VEGFA signalling leading to impaired capillary formation, decreased alveolarization, and decreased vascular density [33].

L-NAME, a potent non-specific inhibitor of nitric oxide synthase (NOS), has been used by other researchers in pregnant rabbits, with confirmation of associated decreased vascularisation and blood flow through the placenta [6,11]. However, the active metabolite of L-NAME was detected in both maternal and foetal serum in equal concentrations, thus questioning the relevance of this model as a method of simulating chronic placental-hypoperfusion due to the possibility of the direct effect of L-NAME itself on the developing foetus, thus decreased NO synthesis in the foetus, and not only the effect of decreased blood flow through the placenta [6]. Thebaud et al. emphasised the need for more extensive animal models to better understand the dysmorphic growth of lung vasculature and the exact mechanisms by which this impacts airway and airspace development [32]. The inhibition of NO pathways has been implicated in the development of multiple lung diseases, including BPD [9]. In a mouse model examining the effects of NO on lung development, eNOS-deficient mice and wild-type mice exposed to L-NAME during mid-to-late gestation exhibited similar changes [34]. To expand the current rabbit model of BPD, the direct effect of L-NAME on the foetus strengthens the model rather than weakens it. However, further investigation into specific pathways and effects related to the timing of L-NAME administration is needed to better understand the pathological disruption of normal lung development.

All the kittens in our study that were exposed to L-NAME showed decreased eNOS in lung tissue on day 7, with no significant difference between those reared in normoxia and those in hyperoxia. VEGFA expression was also reduced in both L-NAME groups, with the LN-Hox lungs being slightly more affected. One can question whether the addition of hyperoxia is necessary, seeing as the LN-Nox kittens had disruption of both eNOS and VEGFA expression; however, only the LN-hox kittens had demonstrable medial thickening of the pulmonary arterioles, thus suggesting that the addition of the second stressor is important. This phenomenon could be explained by eNOS uncoupling, where eNOS produces superoxide (O²-) instead of NO under certain circumstances; however, further study is required to confirm this hypothesis [29].

Further arguments for the necessity of the second hit of hyperoxia become evident when examining the histological findings of the FGR-hox kittens. Although the LN-Nox kittens had a lower lung volume similar to that of the LN-Hox kittens, only the double hit LN-Hox kittens exhibited significant alveolar simplification, consistent with the findings of modern BPD. Similarly, the functional assessment of the double hit LN-Hox kittens showed decreased compliance and increased resistance in the airways and peripheral lung. The LN-Nox kittens exhibited decreased inspiratory capacity and static compliance, although airway and tissue resistance were not significantly affected. This group, however, would still hold value in future therapeutic research, especially to examine the effect of therapies in a milder BPD phenotype. An overview of the phenotypes among the different experimental groups is summarised in S2-S3 Tables in the Supporting Information.

## Conclusion

A double-hit rabbit model combining antenatal FGR with moderate postnatal hyperoxia successfully mimics key features of BPD. The direct disruption of developmental angiogenesis and its contribution to the phenotypes seen should be further investigated. This model is relevant for investigating mechanisms of lung injury and evaluating therapeutic interventions for preterm infants with chronic lung disease or at risk thereof.

## Supporting information

S1 Table Primer sequences used for analysis of gene expression. HPRT: hypoxanthine-guanine phosphoribosyltransferase, housekeeping gene. eNOS: endothelial nitric oxide synthase. VEGFA: vascular endothelial growth factor A.
(DOCX)

S2 Table Summary of phenotypes among different experimental groups. PND7: postnatal day 7, Veh + Nox: vehicle + normoxia, Veh + Hox: vehicle + hyperoxia, LN + Nox: L-NAME + normoxia, LN + Hox: L-NAME + Hyperoxia, eNOS: endothelial nitric oxide synthase, VEGFA: vascular endothelial growth factor A.
(DOCX)

S3 Table Data used for statistical analysis. (XLSX)

## Abbreviations

BPD: bronchopulmonary dysplasia
FGR: foetal growth restriction
L-NAME/LN: N(G)-nitro-L-arginine methyl ester
Veh: vehicle
NO: nitric oxide
GD: gestational day
Nox: normoxia
Hox: hyperoxia
PND: postnatal day
H: tissue elastance
G: tissue damping
Lm: mean linear intercept
Lmw: mean transsectional wall length
VEGFA: vascular endothelial growth factor A
eNOS: endothelial nítric oxide synthase
