# Supplementary material for: Double hit of foetal growth restriction and postnatal hyperoxia alters lung structure and function in a preterm rabbit model of bronchopulmonary dysplasia
Source: PLoS One. 2025 Aug 26;20(8):e0330717. doi: 10.1371/journal.pone.0330717 (PMC12380354; doi:10.1371/journal.pone.0330717)
Supplement: S1 Table — HPRT: hypoxanthine-guanine phosphoribosyltransferase, housekeeping gene. eNOS: endothelial nitric oxide synthase. VEGFA: vascular endothelial growth factor A. (DOCX) [file pone.0330717.s001.docx]

| **Primer** | | **Sequence** |
| --- | --- | --- |
| HPRT | Forward | TGATTAGTGATGATGAACCG |
|  | Reverse | CACACAGAGGGCTACAATG |
| VEGFA | Forward | CTT GCT GCT CTA CCT CCA CC |
|  | Reverse | CTT TGG TCT GCA TTC ACA TTT G |
| eNOS | Forward | ACAGTTACCAGCTCGCCAAA |
|  | Reverse | GCTCATTCTCCAGGTGCTTC |
