# Supplementary material for: Double hit of foetal growth restriction and postnatal hyperoxia alters lung structure and function in a preterm rabbit model of bronchopulmonary dysplasia
Source: PLoS One. 2025 Aug 26;20(8):e0330717. doi: 10.1371/journal.pone.0330717 (PMC12380354; doi:10.1371/journal.pone.0330717)
Supplement: S2 Table — PND7: postnatal day 7, Veh + Nox: vehicle + normoxia, Veh + Hox: vehicle + hyperoxia, LN + Nox: L-NAME + normoxia, LN + Hox: L-NAME + Hyperoxia, eNOS: endothelial nitric oxide synthase, VEGFA: vascular endothelial growth factor A. (DOCX) [file pone.0330717.s002.docx]

| **Characteristic** | **Veh+Nox** | **Veh+Hox** | **LN+Nox** | **LN+Hox** |
| --- | --- | --- | --- | --- |
| *Growth restricted at birth* | No | No | Severe | Severe |
| *Growth restricted at PND7* | No | Mild | Moderate | Moderate |
| *Lung function: compliance* | Normal | Reduced | Reduced | Reduced |
| *Lung function: resistance* | Normal | Mildly reduced | Normal | Moderately reduced |
| *Alveolarization* | Normal | Simplification | Normal | Simplification |
| *Vascular remodelling* | No | Yes | No | Yes |
| *Gene expression: eNOS* | Normal | Moderately reduced | Severely reduced | Severely reduced |
| *Gene expression: VEGFA* | Normal | Mildly reduced | Moderately reduced | Severely reduced |
